# Food and Feed Safety of NS-B5ØØ27-4 Omega-3 Canola (*Brassica napus*): A New Source of Long-Chain Omega-3 Fatty Acids

**DOI:** 10.3389/fnut.2021.716659

**Published:** 2021-09-30

**Authors:** Susan C. MacIntosh, Megan Shaw, Michael Connelly, Zhuyun June Yao

**Affiliations:** ^1^MacIntosh and Associates, Inc., White Bear Lake, MN, United States; ^2^Nuseed Pty Ltd., Laverton North, VIC, Australia; ^3^Nuseed Nutritional US Inc., West Sacramento, CA, United States

**Keywords:** DHA canola, NS-B5ØØ27-4, omega-3, long-chain polyunsaturated fatty acid (LCPUFA), nutrition, food safety, feed safety, sustainability

## Abstract

DHA canola, a genetically engineered *Brassica napus* (OECD Unique Identifier NS-B5ØØ27-4), has been developed as one of the first land-based production systems for omega-3 long-chain polyunsaturated fatty acids (LCPUFA), whose health benefits are well-established. Yet, the marine sources of these nutrients are under high pressures due to over-fishing and increasing demand. DHA canola is a plant-based source for these essential fatty acids that produces a high level of docosahexaenoic acid (DHA). This terrestrial system allows for sustainable, scalable and stable production of omega-3 LCPUFA that addresses not only the increasing market demand, but also the complex interplay of agriculture, aquaculture, and human nutrition. The vector used to produce the desired oil profile in DHA canola contains the expression cassettes of seven genes in the DHA biosynthesis pathway and was specifically designed to convert oleic acid to DHA in canola seed. The characterization and safety evaluation of food and feed produced from DHA canola are described and supported by a detailed nutritional analysis of the seed, meal, and oil. Aside from the intended changes of the fatty acid profile, none of the other compositional analytes showed biologically meaningful differences when compared to conventional canola varieties. In addition, the meal from DHA canola is compositionally equivalent to conventional canola meal. Further evidence of nutritional value and safety of DHA canola oil have been confirmed in fish feeding studies. Given that most human populations lack sufficient daily intakes of omega-3 LCPUFA, a dietary exposure assessment is also included. In conclusion, the results from these studies demonstrate it is safe to use products derived from DHA canola in human foods, nutraceuticals, or animal feeds.

## Introduction

Fatty acids (FA) are carboxylic acids with long-chain hydrocarbon side groups, typically found in esterified form as the major component of lipids. Lipids and FA are sources of energy, most are integral in cell membranes, and indispensable for processing biological and biochemical information. Omega-3 fatty acids (ω3 FA) are essential, being required for human health and obtained primarily from the diet. ω3 FA are a group of polyunsaturated FA (PUFA) that are important for numerous biological functions, including muscle activity, blood clotting, digestion, fertility, cell division and growth, and reducing inflammation (1). The three principal ω3 FA most studied are alpha-linolenic acid (ALA), eicosapentaenoic acid (EPA), and docosahexaenoic acid (DHA). DHA is a primary structural component of many human tissues and is important for brain development and function (2, 3). ω3 FA also play a critical role in the development and function of the central nervous system (3, 4). While the health benefits of ω3 FA are well-known, few people consume enough of these essential nutrients, especially LCPUFAs (5). DHA canola can help address this health concern by providing a safe, easily scalable and sustainable source of ω3 FA.

The main source of EPA and DHA is seafood, including fish (e.g., salmon, tuna, and trout), and shellfish. But marine stocks are diminishing as a result of climate effects and over-fishing. Commonly used dietary supplements include fish oil (which provides EPA and DHA), flaxseed oil (which provides ALA), and algal oil-based supplements that provide a vegetarian source of DHA. Supplementing animal feeds with ω3 FA can result in foods like salmon with increased amounts of ω3 FA (6). Direct supplementation, usually with algal sourced ω3 FA, are now common for cereals, breads, infant foods, condiments, and even pet foods (7). Alternative sustainable sources of EPA and DHA are needed to meet increasing demand. Numerous efforts have been made to develop transgenic oilseed plants that produce omega-3 long-chain polyunsaturated fatty acids (ω3 LCPUFA), including DHA (8, 9).

Canola is an oil crop that is grown on about 44 million hectares globally^1^ and is an excellent production platform for DHA given its 40–45% seed oil content. The genetically engineered DHA canola (OECD Unique Identifier NS-B5ØØ27-4) expresses a modified fatty acid pathway that has been developed as one of the first land-based novel production systems for ω3 LCPUFA. The oil profile was modified through the introduction of seven microalgal and yeast genes that provide a step-by-step conversion of endogenous canola oleic acid (OA) to fish-like levels of DHA in the seed. DHA canola oil will be available as an alternative source of ω3 FA in existing markets for fish oils or established markets for ω3 oils.

Agricultural biotechnology has been widely adopted by growers demonstrating that the technology brings exceptional benefits for effective control of crop pests, reduced inputs, and increased cost savings. The more recent wave of agricultural biotechnology products includes output traits that provide direct benefits to the consumer enhancing the nutritional quality of the food supply. A full characterization of the modified crop, or crop “event,” is typically the first step in any safety assessment. The molecular construction of the introduced genetic vector is described and then confirmed with a molecular analysis of the crop rDNA using modern sequencing techniques coupled with *in silico* bioinformatics analysis to ensure the integrity of the intended introduced genes and their expressed protein products. The newly expressed proteins are characterized with extensive physio-chemical methods, including evaluations of protein stability. The modified crop is evaluated for agronomic, phenotypic, nutritional, and toxicology qualities along with specific data collected on the level of expression of the introduced genes and proteins. Together, this strategy provides a very high level of confidence in the safety of crops developed through modern agricultural biotechnology (10).

**Figure 1:**
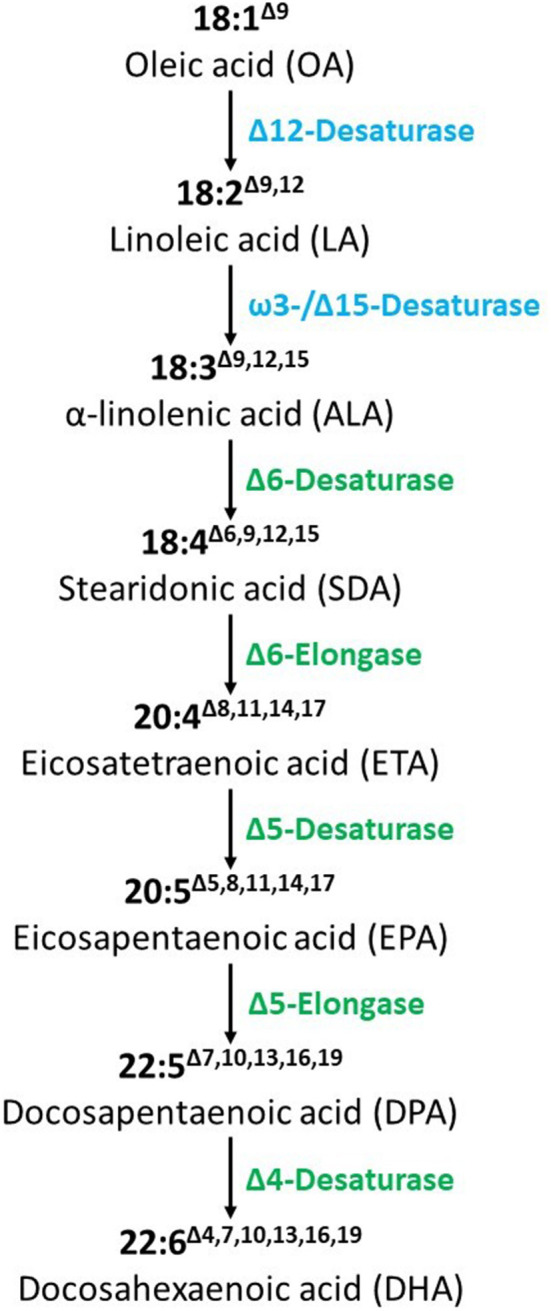
Biosynthesis of DHA from OA in DHA canola. The synthesized genes coding for these enzymes based on yeast (blue) or algal (green) sequences were constructed in a T-DNA vector under the control of seed-specific promoters.

The seven FA desaturases and elongases were introduced to convert OA to DHA (Figure 1). Full characterizations of the vector construct, T-DNA insertion site, copy number, lack of vector backbone, and genetic stability were reported previously (9). DHA canola contains two T-DNA inserts that are required to produce the desired trait (9). The proteins are expressed in a targeted tissue-specific manner, only in developing and mature seed (11). *In vitro* protein digestibility has been documented (12). Bioinformatics analyses showed these introduced proteins have no similarity with known allergens, toxins, or antinutrients. DHA canola has been tested and grown across thousands of acres in a wide range of existing canola production areas in Australia, United States, and Canada without any observed adverse environmental effects, showing standard growth characteristics unchanged from the parental control, AV Jade. In summary, numerous studies, including molecular, biochemical, genetic stability, bioinformatics, nutritional, phenotypic and agronomic characteristics, and animal studies have confirmed the food, feed, and environmental safety of DHA canola leading to regulatory approvals in Australia^2^, Canada^3^, and the USA^4^. Recently, the US FDA recognized Nutriterra® Total Omega-3, a commercial formulation of DHA canola oil as a New Dietary Ingredient allowing use as a nutraceutical supplement in the US^5^. This paper focuses on the food and feed safety aspects, including compositional and nutritional analysis, bioinformatic evaluations, and dietary exposure assessment.

**Figure 2:**
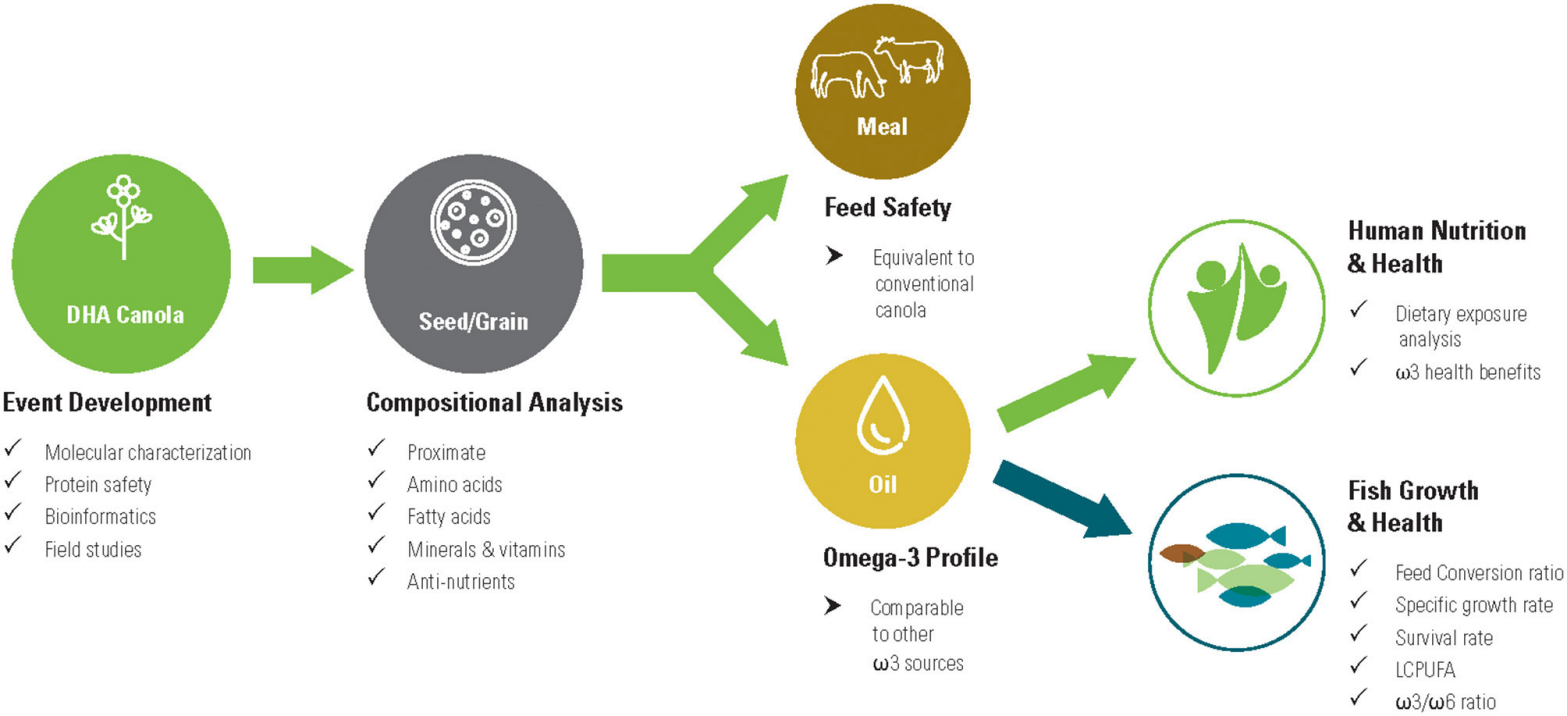
Key studies and safety evaluations of DHA canola for food and feed uses.

DHA canola oil offers a novel alternative to fish oil, a terrestrial source that is easily scalable and sustainable. The substitution of DHA canola oil for fish oil across many sectors (e.g., animal feed, nutritional supplements, food additives) requires a nutritional analysis and safety evaluation. The objective of the current study is to analyze both canola grain and meal in order to fully evaluate the nutritional qualities of the novel fatty acid profile expressed in DHA canola. This allows comparisons of DHA canola oil with common ω3 oil sources currently in the food and feed supply. It also demonstrates that DHA canola is substantially equivalent to conventional canola except for the intended FA profile modification. Finally, fish feeding studies confirmed the nutritional equivalence of DHA canola. Figure 2 summarizes the key studies and safety evaluations for DHA canola.

## Materials and Methods

For any new biotechnology trait, data, and plant samples are collected from field trials planted across a wide range of environmental conditions. Prior to government environmental approvals, all field trials are conducted under permit.

### Canola Grain Production

DHA canola grain was produced in multiple field trials in 2015 to produce grain for nutritional and protein analysis. Approximately 400 g DHA canola grain from eight 2015 locations in major Australian canola growing regions were collected for compositional analysis. Each trial was designed as a randomized complete block consisting of five replicates with the DHA canola event, a parental non-transgenic canola and seven reference canola varieties.

### Canola Meal Production

Harvested DHA canola grain and the parental non-transgenic canola control grain from two 2015 field trials (~2 kg for each crush) were processed into meal for compositional analysis. The DHA canola grain samples were crushed separately at the Commonwealth Scientific and Industrial Research Organisation (CSIRO) Agriculture and Food facility (Werribee, VIC, Australia) using standard canola expeller-pressed crushing, and a portion of the resultant press cake was solvent extracted to remove residual oil to produce hexane-extracted meal^6^. The two parental canola control replicates were also crushed separately to produce expeller-pressed meal and hexane-extracted meal as control samples.

### Compositional Analysis

Compositional analysis can determine if a modified crop is nutritionally equivalent to the conventional counterpart except for the intentionally introduced changes. Since DHA canola expresses a modified FA pathway, the compositional assessment also characterizes and confirms the introduced trait with a detailed FA profile analysis.

Sampled grain was shipped at ambient temperature to the laboratory for analysis (Eurofins Nutritional Analysis Center, Des Moines, IA). The comparative analysis was based on a standard set of analytes, as described in the OECD Consensus Document on compositional considerations for low erucic acid rapeseed (canola; *Brassica napus*) (13). Compositional analysis of grain and meal samples included proximates, FA, amino acids, vitamins, minerals, phytosterols, and key anti-nutrients. Assay specifics, such as methodology, units, and limits of quantitation for each analyte are listed in Supplementary Tables A, B for grain and meal, respectively. DHA canola was compared to its non-transgenic canola parental control along with several commercial reference canola varieties. The ILSI Composition Database^7^ was used to provide additional reference ranges.

### Statistical Analysis

Descriptive statistics using SAS (v9.4) to summarize each analyte for each canola variety, including mean, min, max and standard deviation were determined. A linear mixed model with genotype as a fixed factor and site as a random factor was used to compare the DHA canola and the parental non-transgenic canola control. For each site and each analyte the difference between the DHA canola and parental control was estimated after conducting an ANOVA analysis. Statistics are not reported when analyte values were below the limit of detection (LOD), or when more than 33% of the samples had values below the limit of quantification (LOQ). When a statistically significant difference between the DHA canola and parental variety was identified, further comparisons were made to the range of the reference varieties and to ILSI Composition database (see text footnote 7) or OECD (13) values. When the DHA canola values were within the natural variation of that analyte (reference, ILSI, and/or OECD), no biological significance can be identified.

### Bioinformatics Analysis

Bioinformatics or *in silico* analyses were used to evaluate the genetic sequences across the newly inserted T-DNA and at each junction with interrupted endogenous canola sequences to determine if there were any potential unintended expressions of new open reading frames (ORFs). The entire T-DNA insert in each locus was also evaluated for ORF prediction for sequences representing at least 30 or more contiguous amino acids.

Bioinformatic evaluations were then performed to investigate possible similarities with toxins or allergens using the NCBI Entrez Protein^8^ database as well as the AllergenOnline.org database (Version 18), with methods previously described (14, 15). BLASTP searches of the NCBI Entrez Protein database were done to compare any putative peptide sequences against all protein sequences to determine the prevalence of common homologs using keywords.

## Results

### Compositional Analysis of DHA Canola Grain

The levels of proximates and FA are described in Tables 1, 2, respectively. The levels for glucosinolates, phytosterols and phenolics, amino acids, minerals, and vitamins are described in Supplementary Tables C–G. More than 110 composition analytes were measured or calculated. Additional composition natural variation values were reported from OECD (13) when no data was available from the ILSI Composition database (Supplementary Table D).

**Table 1:** Proximate analysis of DHA canola grain (% DW unless noted otherwise).

**Analyte**	**Test material**	**Mean**	**Std dev**	**Min**	**Max**	**Reference range**	ILSI db^*^ range
Acid detergent fiber	Parental control	11.2	1.4	8.7	14.6	8.6–16.5	8.9–42.3
	DHA canola	11.4	1.4	9.6	16.6		
Ash	Parental control	3.7	0.5	2.9	4.5	2.7–4.5	2.8–8.7
	DHA canola	3.8	0.4	3.1	4.6		
Carbohydrates	Parental control	33.0	2.3	27.0	37.0	27.3–42.3	17.7–47.4
	DHA canola	35.4	2.0	31.4	38.4		
Crude fat	Parental control	33.2	2.9	27.8	39.5	25.5–42.1	24.6–55.2
	DHA canola	30.5	2.7	25.8	35.9		
Crude fiber	Parental control	14.9	1.9	10.1	17.5	10.9–22.6	11.2–37.8
	DHA canola	14.7	2.0	11.3	17.9		
Neutral detergent fiber	Parental control	15.6	1.6	12.6	18.8	12.1–21.8	10.9–53.7
	DHA canola	15.6	1.1	13.6	18.1		
Protein	Parental control	30.1	1.2	26.9	32.2	23.5–32.1	15.6–35.7
	DHA canola	30.4	1.2	27.5	32.5		
Moisture %FW – fresh weight	Parental control	7.9 %FW	0.3	7.4	8.4	6.6–8.6 %FW	3.2–34.6 %FW
	DHA canola	8.2 %FW	0.3	7.6	8.7		

* *ILSI composition database (db), Version 7*.

**Table 2:** FA analysis of DHA canola grain (% of total FA).

**Analyte**	**Test material**	**Mean**	**Std dev**	**Min**	**Max**	**Reference range**	ILSI db^*^ range
C14:0 (Myristic)	Parental control	0.08	0.01	0.07	0.09	0.06–0.10	0.04–0.09
	DHA canola	0.08	0.01	0.07	0.09		
C16:0 (Palmitic)	Parental control	4.31	0.11	4.09	4.58	3.62–4.82	3.55–5.70
	DHA canola	4.50	0.09	4.37	4.69		
C16:1 (Palmitoleic)	Parental control	0.27	0.01	0.25	0.30	0.21–0.34	0.16–0.40
	DHA canola	0.29	0.01	0.27	0.32		
C17:0 (Margaric)	Parental control	0.05	0.01	0.04	0.06	0.04–0.06	0.03–0.14
	DHA canola	0.05	0.01	0.04	0.06		
C17:1 (Ginkgolic)	Parental control	0.06	0.00	0.05	0.07	0.05–0.08	0.04–0.16
	DHA canola	0.04	0.00	0.04	0.05		
C18:0 (Stearic)	Parental control	2.21	0.08	2.05	2.34	1.41–2.26	1.50–2.77
	DHA canola	2.15	0.08	2.02	2.46		
C18:1 n-9 (OA)	Parental control	57.07	1.48	54.59	59.91	49.16–72.68	NR
	DHA canola	42.03	2.43	37.23	47.38		
C18:1 total	Parental control	59.82	1.44	57.40	62.60	51.93–74.36	53.19–69.45
	DHA canola	45.00	2.38	40.44	50.28		
C18:2 n-6 (LA)	Parental control	19.34	0.83	16.60	20.58	11.59–23.26	NR
	DHA canola	8.50	0.24	8.04	9.08		
C18:3 n-3 (ALA)	Parental control	11.18	0.74	9.96	12.62	3.90–12.08	NR
	DHA canola	21.04	1.08	18.81	22.87		
C18:3 total	Parental control	11.28	0.77	10.02	12.73	3.93–12.19	5.79–12.09
	DHA canola	22.20	1.14	19.81	24.19		
C18:4 n-3 (SDA)	Parental control	0.03	0.03	0.01	0.17	0.03–0.31	NR
	DHA canola	2.52	0.29	1.99	3.22		
C20:0 (Arachidic)	Parental control	0.48	0.01	0.46	0.50	0.42–0.73	0.49–0.86
	DHA canola	0.59	0.01	0.57	0.62		
C20:1 n-9 (Gondoic)	Parental control	0.95	0.02	0.90	1.04	0.88–1.59	1.00–1.82
	DHA canola	1.18	0.03	1.13	1.25		
C20:2 n-6 (Eicosadienoic)	Parental control	0.06	0.01	0.05	0.07	0.05–0.19	0.04–0.86
	DHA canola	0.09	0.01	0.08	0.10		
C20:3 n-3 (ETE)	Parental control	0.05	0.03	0.03	0.13	0.02–0.10	NR
	DHA canola	0.58	0.05	0.46	0.67		
C20:4 n-3 (ETA)	Parental control	0.07	0.05	0.03	0.26	0.03–0.12	NR
	DHA canola	1.14	0.06	1.00	1.29		
C20:5 n-3 (EPA)	Parental control	0.05	0.02	0.04	0.08	0.04–0.08	NR
	DHA canola	0.44	0.04	0.32	0.52		
C22:0 (Behenic)	Parental control	0.19	0.01	0.18	0.20	0.18–0.39	0.19–0.46
	DHA canola	0.25	0.01	0.24	0.27		
C22:5 n-3 (DPA)	Parental control	0.07	0.04	0.03	0.20	0.03–0.15	NR
	DHA canola	1.05	0.09	0.80	1.23		
C22:6 n-3 (DHA)	Parental control	0.15	0.28	0.03	1.55	0.03–1.34	NR
	DHA canola	8.38	0.81	6.50	10.30		
C24:0 (Lignoceric)	Parental control	0.10	0.01	0.09	0.11	0.10–0.21	0.09–0.26
	DHA canola	0.09	0.01	0.08	0.10		
C24:1 n-9 (Nervonic)	Parental control	0.10	0.01	0.08	0.11	0.10–0.17	0.08–0.40
	DHA canola	0.06	0.00	0.05	0.07		
FA total (%DW – dry weight)	Parental control	29.91	1.95	26.27	34.07	23.94–35.78	24.6–55.2 %DW^**^
	DHA canola	27.26	1.88	22.23	31.02		
EPA + DPA + DHA	DHA canola	9.86	NR	7.62	12.02	NR	NR

* *ILSI composition database (db), Version 7; NR, not reported.*

** *ILSI FA total value is taken from the Crude Fat value from the Proximate analysis*.

No biological differences between DHA canola grain and the parental grain were identified for the analytes measured except for the intended FA modification. Small quantities of DHA were observed in the parental grain, due to the mixed plot design of the field trial.

Canola was originally produced from rapeseed through a traditional breeding program to reduce the nutritionally undesirable components of glucosinolates and erucic acid. The successful reduction in the levels of glucosinolates (30 μmol/g)^9^ and erucic acid (<2% of total FA) allowed canola to be granted GRAS status by US FDA in 1985. For compositional analysis, the means of glucosinolates (Supplementary Table C) were summed and the totals were 12.1 and 11.9 μmol/g for the DHA canola and parental control, respectively, lower than the 30 μmol/g threshold. Erucic acid (C22:1 n-9) was below the LOQ for DHA canola, and the parental control was well below 0.1%. DHA canola has values well below the canola thresholds for glucosinolates and erucic acid, ensuring its nutritional value and no increased risks to people and animals.

Because DHA canola expresses seven FA pathway enzymes, it is not surprising that many of the FA levels were significantly different from conventional canola, including reduced levels of OA (37–47%) and linoleic acid (LA) (8–9%), and increased levels of ALA (19–23%). Several ω3 fatty acids are newly produced, including stearidonic acid (SDA) (2–3%), EPA (0.3–0.5%), docosapentaenoic acid (DPA) (0.8–1.2%), and most notably, DHA (6.5–10.5%). Smaller quantities of eicosatrienoic acid (ETE) and eicosatetraenoic acid (ETA) are also present in DHA canola with the total ω3 in DHA canola oil ~33–35%.

Differences were found in several of the phytosterol analytes (Supplementary Table D). Total phytosterols, both FW and DW calculations, were slightly higher for DHA canola when compared to either the parental variety or the reference range. However, DHA canola values fell within the range reported by OECD (13), and a small elevation is nutritionally beneficial since phytosterols play an important role in the reduction of cholesterol and improved heart health (16).

There were very few differences observed for the amino acids, minerals, or vitamins levels when comparing DHA canola to its parental variety (Supplementary Tables E–G, respectively). In every case, the ranges overlapped and the DHA canola analyte means fell within the reference or the ILSI composition database ranges.

### Compositional Analysis of DHA Canola Meal

Canola is processed by crushing into oil and meal fractions. The resultant meal fraction undergoes further processing by solvent extraction to remove any remaining oil, leaving the defatted meal with only trace amounts of oil. DHA canola will be processed in a similar manner, albeit under an identity preservation process, to extract the value of the oil. Conventional solvent extracted canola meal is an excellent source of protein (36–44%) and fiber for livestock, poultry and fish (13). Incorporation rates of 5–30% are routinely used depending on the species. Compositional analysis of meal samples for proximates and FA are provided in Tables 3, 4, respectively. The limits of quantitation and units are listed in Supplementary Table B.

**Table 3:** Proximate of expeller-pressed and hexane-extracted DHA canola meal (% DW).

**Analyte**	**Test material**	**Mean (% DW)**	
		**Expeller-pressed meal**	**Hexane-extracted meal**
Acid detergent fiber	Parental control	15.8	21.1
	DHA canola	17.0	18.2
Ash	Parental control	5.0	6.3
	DHA canola	5.0	5.9
Carbohydrates	Parental control	30.9	39.2
	DHA canola	35.1	40.6
Crude fat	Parental control	21.4	0.7
	DHA canola	15.7	0.4
Crude fiber	Parental control	8.6	10.4
	DHA canola	8.7	9.2
Neutral detergent fiber	Parental control	24.5	31.6
	DHA canola	23.3	28.3
Protein	Parental control	42.6	54.6
	DHA canola	44.2	53.0

**Table 4:** FA of expeller-pressed and hexane-extracted DHA canola meal (% of total FA).

**Analyte**	**Test material**	**Mean (% FA)**	
		**Expeller-pressed meal**	**Hexane-extracted meal**
C14:0 (Myristic)	Parental control	0.02	<LOQ
	DHA canola	0.01	<LOQ
C16:0 (Palmitic)	Parental control	0.86	0.04
	DHA canola	0.69	0.02
C16:1 (Palmitoleic)	Parental control	0.08	<LOQ
	DHA canola	0.06	<LOQ
C18:0 (Stearic)	Parental control	0.39	<LOQ
	DHA canola	0.31	<LOQ
C18:1 n-9 (OA)	Parental control	10.11	0.21
	DHA canola	5.17	0.06
C18:2 n-6 (LA)	Parental control	3.64	0.12
	DHA canola	1.18	0.01
C18:3 n-3 (ALA)	Parental control	1.99	0.04
	DHA canola	3.03	0.02
C18:4 n-3 (SDA)	Parental control	0.02	<LOQ
	DHA canola	0.44	<LOQ
C20:0 (Arachidic)	Parental control	0.09	<LOQ
	DHA canola	0.08	<LOQ
C20:1 n-9 (Gondoic)	Parental control	0.17	<LOQ
	DHA canola	0.15	<LOQ
C20:2 n-6 (Eicosadienoic)	Parental control	0.07	<LOQ
	DHA canola	0.01	<LOQ
C20:3 n-3 (ETE)	Parental control	<LOQ	<LOQ
	DHA canola	0.09	<LOQ
C20:4 n-3 (ETA)	Parental control	0.01	<LOQ
	DHA canola	0.18	<LOQ
C20:5 n-3 (EPA)	Parental control	<LOQ	<LOQ
	DHA canola	0.07	<LOQ
C22:0 (Behenic)	Parental control	0.04	<LOQ
	DHA canola	0.04	<LOQ
C22:5 n-3 (DPA)	Parental control	0.01	<LOQ
	DHA canola	0.17	<LOQ
C22:6 n-3 (DHA)	Parental control	0.06	<LOQ
	DHA canola	1.31	<LOQ
C24:0 (Lignoceric)	Parental control	0.02	<LOQ
	DHA canola	0.01	<LOQ

Protein, fat, and fiber are the key indicators of livestock feed quality (Table 3) along with amino acids and digestibility. As expected, crushing resulted in a drastic reduction of FA in both DHA canola and parental variety meal samples, and the FA profiles matched those observed in grain (Table 4). In all cases, the amount of FA in hexane-extracted meal were reduced by 95% of that measured in expeller-pressed meal. The amount measured for all glucosinolates in both expeller-pressed or hexane-extracted meals ranged from 15.6 to 19.6 μmol/g, below the canola standard (30 μmol/g) (see text footnote 9). Otherwise, the composition of meal reflected what was found in the analysis of the whole grain.

### Nutritional Composition and Comparators

The parental variety was used as the conventional control to support the safety assessment of DHA canola. Several commercial reference varieties were included in the nutritional assessments to provide a range of comparative values for each characteristic. These varieties represent a range of maturities, phenotypes, yield potential, and disease resistance, reflecting natural variability within commercial varieties grown in Australia.

As DHA canola was designed to produce ω3 LCPUFA, including EPA, DPA, and especially DHA, no single comparator is adequate, although many different food sources of ω3 FA are commonly consumed (Table 5). ω3 FA are found in microalgae, ocean fish, crustacean, and some food crops, such as flaxseed that contains medium chain ω3 FA. Many algal sources contain high levels of DHA, for example, some *Schizochytrium* species produce up to 37.5% DHA of total FA, which are well above levels found in menhaden, anchovy, salmon, or krill oils (5–26% DHA of total of FA). As shown in Table 5, the levels and forms of the introduced ω3 PUFA in DHA canola oil are comparable and consistent with ω3 PUFA already consumed in human diets.

**Table 5:** ω3 FA profile of DHA canola oil and multiple ω3 oil comparators (% of total FA).

**Fatty acid**	**DHA canola oil**	**Flaxseed oil^**a**^**	**Menhaden oil^**b**^**	**Anchovy oil^**b**^**	**Salmon oil (farmed)^**b**^**	***Schizochytrium* oil^**c**^**	**Krill oil^**b**^**
C18:3 n-3 (ALA)	18.8–22.9	43.8–70.0	ND-2.0	ND-7.0	3.0–6.0	NR	0.1–4.7
C18:4 n-3 (SDA)	1.9–3.2	NR	1.5–3.0	ND-5.0	0.5–1.5	Trace-0.8	1.0–8.1
C20:4 n-3 (ETA)	1.0–1.3	NR	NR	ND-2.0	0.5–1.0	0.8–0.9	NR
C20:5 n-3 (EPA)	0.3–0.5	NR	12.5–19.0	5.0–26.0	2.0–6.0	2.0–3.2	14.3–28.0
C22:5 n-3 (DPA)	0.8–1.2	NR	2.0–3.0	ND-4.0	1.0–2.5	NR	ND-0.7
C22:6 n-3 (DHA)	6.5–10.3	NR	5.0–11.5	4.0–26.5	3.0–10.0	32.5–37.5	7.1–15.7

*NR, not reported*.

a *CODEX 2019 “CX/FO 19/26/8”.*

b *CODEX 2017 “CXS 329-2017”.*

c *US FDA GRN 137*.

### Fish Feeding Trials

The growing aquaculture industries also need access to cost effective sources of ω3 FA for fish nutrition. Aquaculture is the major consumer for fish oil since marine fish and shrimp require certain levels of EPA and DHA for efficient growth and survivability. Additionally, the presence of ω3 oils in aquaculture feeds results in deposition of these FA in fish and shrimp flesh, providing this important dietary nutrition benefit for human consumption.

Experiments on the survival, growth, and whole-body FA content of young Atlantic salmon (*Salmo salar*) growing in fresh water were conducted in Norway and Australia to assess the safety and effectiveness of oil derived from DHA canola as a partial replacement of fish oil (17). Growth of Atlantic salmon fry from ~1–2 g to 20–25 g was equal across all dietary treatments and at both locations. Survival rate was >94% in all cases, with no difference observed between the diets. FA from DHA canola oil were incorporated into fish in equal concentrations to those from fish oil.

In addition, three large-scale, on-farm trials were conducted in Chile to evaluate the performance of DHA canola oil as a partial replacement of fish oil in Atlantic salmon diets. In each trial, a standard commercial salmon diet (control) was compared with a test diet formulated such that 30–60% of the fish oil was replaced with DHA canola oil, also known commercially as Aquaterra®. Total EPA+DHA in the diet was held constant for each trial.

**Figure 3:**
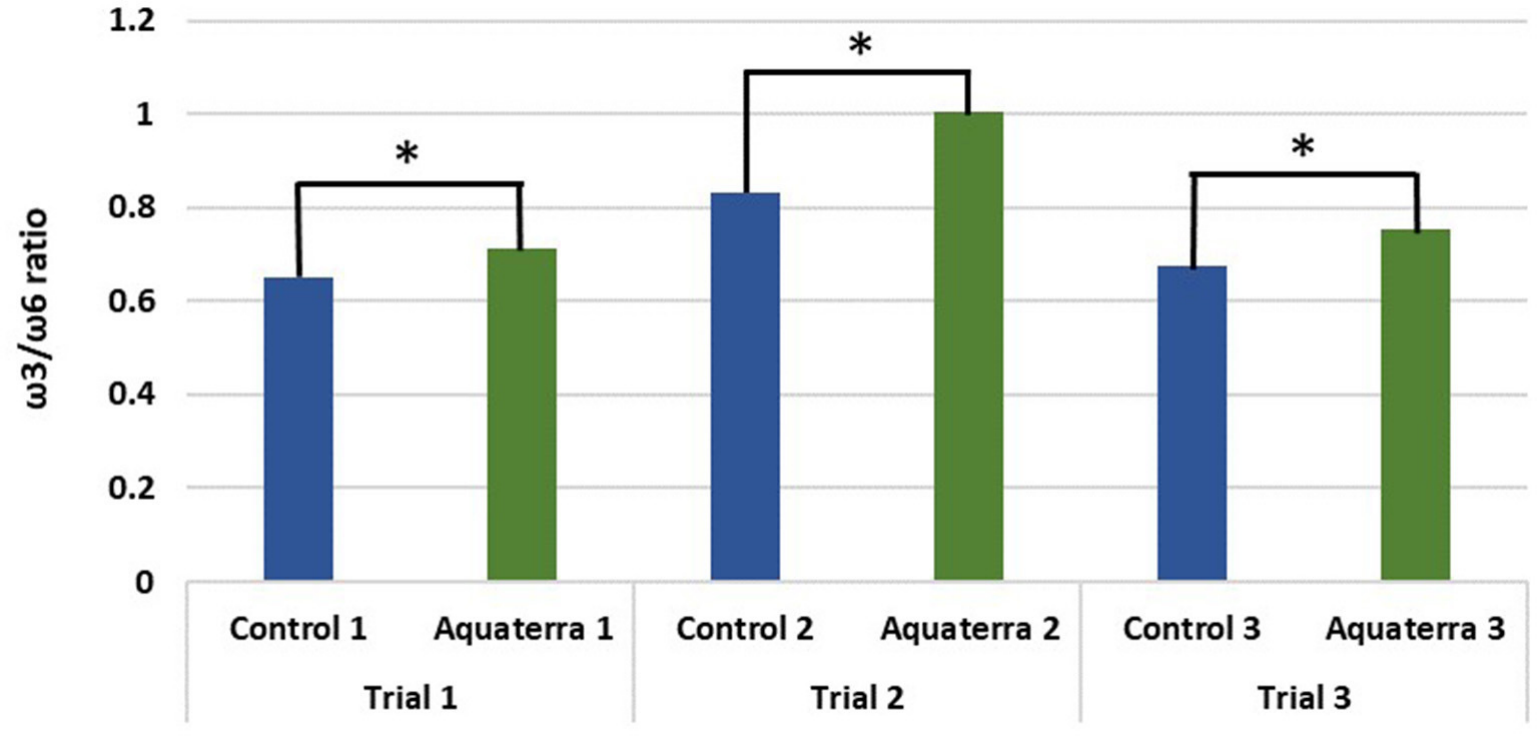
ω3/ω6 ratio in filets raised on the Aquaterra Omega-3 oil and control diets. *Statistically significant difference with 95% confidence level.

Feed conversion ratio (FCR), specific growth rate (SGR), and survival rate were recorded as the most relevant productive aspects to evaluate growth. There were no differences in weight gain, SGR, and FCR between the control and Aquaterra diets (Table 6). Mortality was consistently 1.5–1.9% lower in fish fed the Aquaterra diet. DHA content of filets was higher in fish fed the Aquaterra diet. Total EPA+DHA levels fell within the normal range for farm-raised salmon. ALA contributed to a high ω3 content in Aquaterra diets and resulted in a higher ω3/ω6 FA ratio in filets from fish receiving the Aquaterra diets (Figure 3). A detailed organoleptic analysis of fish from one trial indicated no differences in taste, odor, or quality parameters in fish raised on the control or Aquaterra diets (6).

**Table 6:** Summary of production performance in three industrial-scale fish trials.

**Production variables**	**Trial 1**		**Trial 2**		**Trial 3**	
	**Control**	**Aquaterra**	**Control**	**Aquaterra**	**Control**	**Aquaterra**
Initial weight (g)	1,623	1,585	1,240	1,134	158	131
Gained weight (g)	3,703	3,680	4,994	4,889	5,593	5,552
SGR	0.48	0.48	0.52	0.56	0.81	0.83
SFR	0.62	0.61	0.72	0.76	1.07	1.12
FCRb	1.28	1.28	1.40	1.37	1.43	1.40
Survival	93.61	95.1	89.28	91.18	88.86	90.73

*SGR, specific growth rate; SFR, specific feed rate; FCRb, biological feed conversion ratio*.

These results confirm that oil from DHA canola is a safe and effective substitution for fish oil in Atlantic salmon feed, during the most sensitive stages in their life cycle as well as during their full growth cycle. Supplementing animal feeds with ω3 oils sourced mostly from fish is a critical practice for the industry. DHA canola oil will provide an economic and sustainable alternative.

### Bioinformatics—Junction ORF Analysis, T-DNA Insert ORF Analysis

Five junction sequences were identified from the molecular characterization of DHA canola: two on either side of the inserts and one on the T-DNA linking two eight-gene sets in the palindrome structure of one of the inserts. The length of putative peptides ranged from 12 to 52 amino acids across the five junctions and both inserts. There were no relevant matches for the ORF junction analysis. Across the T-DNA inserts, several sequences were identified using the start-to-stop approach (Supplementary Table H). This approach only identified peptides that were substantially long and might have a suspected match with an allergen.

The ORF34 sequence matched to the 2S albumin of black walnut, *Juglans nigra* accession #31321942 (Supplementary Table H). The protein is unlikely to be expressed because the four significant gaps demonstrate that similarity to IgE binding epitopes would not be replicated, if this ORF were to be translated. Other alignments to potential allergens or toxins were not relevant as the ORFs had closer percent identity matches to the gene-source donors compared to other matched segments as shown by the “No keyword” alignments. The seven proteins needed for production of DHA and the PAT protein were derived from the gene-source donors.

## Discussion

### Summary of Event Development

The DHA canola event was produced by the introduction of seven biosynthetic genes and the *pat* gene, using the binary vector (pJP3416_GA7-ModB). The introduced genes were synthesized, codon optimized and verified to ensure the amino acid sequence of the proteins expressed in DHA canola were identical to the sequences of microalgal/yeast source proteins. Several vectors were constructed and tested during the development of DHA canola to enhance the DHA content level by increasing the enzymatic efficiency of each enzyme. The binary vector was inserted into chromosome A02 as a partial insertion of four intact expression cassettes for Micpu-Δ6D, Pyrco-Δ5E, Pavsa-Δ5D, and Picpa-ω3D, and in chromosome A05 as two complete T-DNA insertions as a head-to-head palindrome. Both inserts were necessary to produce commercially viable levels of DHA in the seed oil. Petrie et al. (9) describes the creation, development and testing of the construct, plant transformation and selection, field testing, and DHA canola seed and oil stability.

### Protein Safety and Expression

The introduced desaturases and elongases are integral membrane proteins, making it extremely difficult to isolate and purify from DHA canola in order to test for equivalency in heterologous systems (18). Nevertheless, safety of these proteins was evaluated by comparing the amino acid (AA) sequence and similarity to other proteins that have a history of safe use, determining the protein expression (11), analyzing *in vitro* digestibility using digestive enzymes (12), and testing thermal degradation. In addition, cloning of each gene was described and the resulting proteins characterized using heterologous expression systems to determine their molecular mass, phylogenetic tree to related enzymes, and functional activity under different conditions (19–21).

The AA sequence homology comparisons demonstrated a wide range of organisms where these types of enzymes were found. While the percent identity match of these enzymes is broad, they function in the same way by adding a double bond (desaturases) or two carbons (elongases) to the FA structure in a very specific manner within each organism. Target and non-target organisms already consume a wide range of sources containing these enzymes, further demonstrating a history of safe use and supporting their safety.

The tissue-specific expression and protein abundance of the seven ω3 pathway enzymes were evaluated using a targeted LC-MRM-MS proteomic methodology that provided absolute quantitation using specifically labeled peptides (11). Results demonstrated that the seven ω3 pathway enzymes were only detected in developing and mature seeds driven by their seed-specific promoters. The expression levels of the seven enzymes ranged from 190 ng/mg total protein for the least abundant protein (Δ6-desaturase) to 5,560 ng/mg protein for the most abundant protein (Δ4-desaturase). The PAT protein was measured in all plant tissues at expression levels from 23 to 390 ng/mg total protein, as would be expected from its constitutive 35Sx2 promoter.

All enzymes were readily digested using a standardized protocol (22) coupled with a LC-MS based proteomic methodology (12). These proteins were also thermally unstable after 30 min at 95°C, a condition of typical canola processing (data not shown).

The safety of the PAT protein is well-established (23). Regulatory authorities around the world have evaluated over 100 transformation events containing PAT and have consistently concluded that the presence of PAT in crops is safe for food and feed (24).

### Compositional Analysis

Aside from the introduced modified FA pathway, DHA canola grain was substantially equivalent to the non-transgenic canola parental control across the 110 analytes. When there were statistical differences identified, the values fell within the natural variation as set by the range of non-transgenic reference varieties, ILSI Compositional database or OECD values. Petrie et al. (9) reports that LCPUFA levels of DHA canola were consistent (10–15% of total FA) across different growing regions, similar to the field locations used in this report. The FA content of DHA canola oil is comparable or lower than many other types of ω3 oils that are commonly consumed (Table 5). Therefore, it is unlikely that DHA canola oil would raise any concerns given the relatively high levels of ALA, SDA, ETA, EPA, DPA, and DHA that are found in flaxseed, fish or algal oils.

Based on the important feed parameters in hexane-extracted meal, the composition of DHA canola meal is not different from conventional canola meal, except for the intended difference in the oil fraction. Meal derived from DHA canola grain is as safe for animal feeds as meal from any commercial canola variety.

Another important nutritional aspect of DHA canola is the favorable ratio of ω3:ω6, which is 4.8:1, as compared to 0.5:1 for the parental variety (9). It was reported that the ω3:ω6 ratio in modern western diets have trended to 1:16 because of an increased dietary consumption of ω6 FA, leading to increases in obesity and inflammation (1, 25). DHA canola oil would offer a healthier FA profile.

### Bioinformatics Analysis

The bioinformatics searches showed no biologically relevant identity between the query sequences and any known toxin, allergen, or protein likely to cause an adverse effect in consumers. These bioinformatic analyses demonstrated that none of the newly expressed proteins or any new ORFs associated with DHA canola have homology with toxicological or allergenic concerns.

### Dietary Exposure Assessment of DHA Canola

DHA canola oil is a suitable replacement of fish oil (Table 5). Direct intake of DHA canola oil is most likely via fortified foods and nutraceuticals. It is not expected that this replacement will result in a change in consumption of ω3-fortified foods and/or levels of ω3 supplementation, although the replacement of an animal origin with a plant-based source may appeal to a significant portion of consumers. No change in dietary exposure in any of the risk categories of human consumption is anticipated.

However, reports indicate that only a small fraction of the population meets the recommended ω3 FA consumption levels. A review of studies reporting blood levels of DHA+EPA indicated very low levels in the populations of the Americas, Europe, Middle East, Africa, Southeast Asia, China, and Australia (5). Only in limited regions, such as Japan, Scandinavia, and indigenous populations, which have not totally adapted to a western diet, are the blood plasma levels of ω3 PUFA considered adequate or high (5). In fact, the World Health Organization (26) and EFSA (27) have recommended a minimum intake of 250 mg/day EPA and DHA combined. The International Society for the Study of Fatty Acid and Lipids (ISSFAL) recommend intakes of 500 mg/day, and the Japanese Ministry of Health, Labour and Welfare recommends a minimum intake of 1,000 mg/day EPA and DHA combined. These organizations all support that higher intakes of EPA and DHA is recommended for most populations.

Defatted DHA canola meal has been shown to be compositionally comparable to other commodity canola meals. As DHA canola meal is compositionally equivalent to other canola meal, it will be utilized in animal diets in the same manner and at the same levels as the existing canola meal commodity. The estimated dietary intake of each introduced protein in DHA canola for livestock and poultry would be generally <0.1% of total daily protein intake/kg BW, given the expression levels observed in Colgrave et al. (11) and the incorporation rates of canola meal in livestock and poultry diets (13).

### Health Benefits of Omega-3 Fatty Acids

Fish and seafood are dietary staples and some of the healthiest foods available. Shrimp and marine fish have a very high nutritional value, provide an excellent source of protein and an array of vitamins, and are one of the best sources of ω3 FA. DHA canola provides a land-based sustainable and scalable system for the production of ω3 oils: ALA, SDA, EPA, DPA, and DHA. The oil will be used in feed, food and nutraceutical applications where ω3 sources are currently used.

ALA has long been established as an essential ω3 FA in the diet with dietary reference intake targets of 1,600 and 1,100 mg/day for men and women (28). The main sources of ALA in the U.S. diet are vegetable oils, particularly canola, and soybean oils. Flaxseed, hemp, and walnut oils also provide rich sources of ALA. DHA canola contains twice as much ALA as conventional canola, providing a great source of this essential nutrient.

DHA, as an essential component of cell membranes of various tissues and organelles in mammals (e.g., nerve, retina, brain, and immune cells) provide many health benefits. Clinical studies have shown that DHA is essential for resolving inflammatory responses, growth and development of infant brains, maintenance of normal brain function in adults, and provides some positive effects on diseases such as hypertension, arthritis, atherosclerosis, depression, thrombosis, and cancers. Because DHA and EPA cannot be efficiently synthesized by infants, young children, and senior citizens, it is particularly important for these individuals to consume these FA from the diet (3). Importantly, an appropriate ratio of ω3 to ω6 FA avoids metabolic problems such as an imbalance of membrane fluidity. DHA canola oil offers an opportunity to improve this ω3:ω6 FA ratio with increased amounts of ALA, SDA, EPA, DPA, and DHA.

## Conclusion

The safety assessment of biotechnology based crops includes a full characterization of the modified crop, as has been accomplished for DHA canola. The molecular and protein aspects have been previously summarized and this report provides a detailed nutritional assessment for both DHA canola grain and meal. *In silico* bioinformatics results demonstrate that the newly introduced proteins have no similarities to known allergens or toxins and that no new unexpected proteins have been expressed surrounding the molecular insertion site.

Nutritionally, DHA canola grain and meal are substantially equivalent to its non-transgenic parental counterpart, except for the introduced and expected changes in the FA profile. Levels of ALA, SDA, ETA, EPA, DPA, and DHA are higher in DHA canola oil as expected, but lower than those found in flaxseed, fish or algal oils, which are commonly consumed without any adverse effects. An estimate of the dietary intake for DHA canola is presented and within the standards set by numerous regulatory authorities. Finally, the benefits of ω3 FA in general and DHA canola oil, specifically, are outlined.

DHA canola is a readily scalable and sustainable land-based production system for ω3 FA. Based on yield and oil content, one hectare of DHA canola has the potential to provide the ω3 oil produced from 10,000 kg fish, demonstrating that it is a cost-effective, safe, and reliable alternative to marine-sourced ω3 oil. Reliable sources of ω3 oil are required by the growing aquaculture industry for the health and welfare of farmed fish. Evidence that higher ω3 LCPUFA levels are associated with a reduced risk of several chronic diseases, including coronary heart disease, suggests that most communities would benefit from higher intakes which this product could provide.

## Data Availability Statement

The original contributions presented in the study are included in the article/Supplementary Material, further inquiries can be directed to the corresponding author/s.

## Author Contributions

SM, MS, and MC made substantial contributions to the design and monitoring of the studies, analysis and interpretation of data, and preparation of the study reports. SM, MS, MC, and ZY did extensive drafting and editing of the manuscript. All authors contributed to the article and approved the submitted version.

## Conflict of Interest

SM was employed by company MacIntosh and Associates Inc. MS was employed by company Nuseed Pty Ltd. MC and ZY were employed by company Nuseed Nutritional US Inc. This research was conducted for Nuseed Pty Ltd., a wholly owned subsidiary of Nufarm Ltd.

## Publisher's Note

All claims expressed in this article are solely those of the authors and do not necessarily represent those of their affiliated organizations, or those of the publisher, the editors and the reviewers. Any product that may be evaluated in this article, or claim that may be made by its manufacturer, is not guaranteed or endorsed by the publisher.

## Footnotes

^1^ http://www.fao.org/faostat/en/?#data/QC

^2^https://www.foodstandards.gov.au/consumer/gmfood/Pages/Food-derived-from-DHA-Canola-LineA1143.aspx; https://www.ogtr.gov.au/gmo-dealings/dealings-involving-intentional-release/dir-155

^3^ https://www.canada.ca/en/health-canada/services/food-nutrition/genetically-modified-foods-other-novel-foods/approved-products.html

^4^https://www.aphis.usda.gov/aphis/ourfocus/biotechnology/permits-notifications-petitions/petitions/petition-status (17-236-01p).

^5^ https://www.fda.gov/food/new-dietary-ingredients-ndi-notification-process/submitted-75-day-premarket-notifications-new-dietary-ingredients

^6^ https://www.canolacouncil.org/oil-and-meal/what-is-canola/how-canola-is-processed/steps-in-oil-and-meal-processing

^7^ https://www.cropcomposition.org/

^8^ http://www.ncbi.nlm.nih.gov/BLAST/

^9^https://www.gipsa.usda.gov/fgis/standards/810canola.pdf and http://www.canolacouncil.org/oil-and-meal/what-is-canola/

## Abbreviations

AA: amino acid
ALA: α-linoleic acid, 18:3^Δ9, 12, 15^
CSIRO: Commonwealth Scientific and Industrial Research Organisation
DHA: docosahexaenoic acid, 22:6^Δ4, 7, 10, 13, 16, 19^
DHA canola, genetically engineered canola: event NS-B5ØØ27-4
DPA: docosapentaenoic acid, 22:5^Δ7, 10, 13, 16, 19^
DGLA: dihomo-γ-linolenic acid
DW: dry weight
EPA: eicosapentaenoic acid, 20:5^Δ5, 8, 11, 14, 17^
ETA: eicosatetraenoic acid, 20:4^Δ8, 11, 14, 17^
ETE: eicosatrienoic acid, 20:3^Δ11, 14, 17^
FA: fatty acid(s)
GLA: γ-linolenic acid
GRAS: generally recognized a safe
Ha: hectares
ILSI: International Life Sciences Institute
LA: linoleic acid, 18:2^Δ9, 12^
LC-MRM-MS: liquid chromatography-multiple reaction monitoring-mass spectrometry
LC-MS: liquid chromatography-mass spectrometry
LCPUFA: long chain (≥C20) polyunsaturated fatty acids
LOD: limit of detection
LOQ: limit of quantification
Micpu-Δ6D: *Micromonas pusilla* Δ6-desaturase
OA, oleic acid: 18:1^Δ9^
ORF: open reading frame
PAT: phosphinothricin-N-acetyltransferase
Pavsa-Δ5D: *Pavlova salina* Δ5-desaturase
Picpa-ω3D: *Pichia pastoris* Δ15-/ω3-desaturase
Pyrco-Δ5E: *Pyramimonas cordata* Δ5-elongase
SDA: stearidonic acid, 18:4^Δ6, 9, 12, 15^
US FDA: United States Food and Drug Administration
ω3: omega-3
ω6: omega-6.
