# Supplementary material for: Food and Feed Safety of NS-B5ØØ27-4 Omega-3 Canola (Brassica napus): A New Source of Long-Chain Omega-3 Fatty Acids
Source: Front Nutr. 2021 Sep 30;8:716659. doi: 10.3389/fnut.2021.716659 (PMC8514783; doi:10.3389/fnut.2021.716659)
Supplement: Supplementary file 1 [file Table_1.docx]

**Food and Feed Safety Evaluation of DHA Canola (*Brassica napus*):**

**A Novel Source of Long-Chain Omega-3 Fatty Acids**

Susan C. MacIntosh^1^, Megan Shaw^2^, Michael Connelly^3^, Zhuyun June Yao^3*^

**Supplementary Material**

**Tables**

**Table A.** Analyte specifics for DHA canola seed compositional analysis

| Parameter | Eurofins Method* | Units | LOQ |
| --- | --- | --- | --- |
| Moisture | MET-PR-005 | % | 0.2% |
| Protein, Crude | MET-PR-002 | % | 0.1% |
| Fat, Crude | MET-LI-001 | % | 0.1% |
| Ash | MET-PR-004 | % | 0.4% |
| Carbohydrates, Calculated | OPS-024 | % | N/A |
| Crude Fiber | MET-PR-003 | % | 0.2% |
| Acid Detergent Fiber | MET-PR-007 | % | 0.3% |
| Neutral Detergent Fiber | MET-PR-008 | % | 0.3% |
| Amino Acids by Acid Hydrolysis | MET-LC-006 | % | Aspartic Acid: 0.02%  Threonine: 0.02%  Serine: 0.01%  Glutamic Acid: 0.01%  Glycine: 0.01%  Alanine: 0.01%  Valine: 0.02%  Isoleucine: 0.02%  Leucine: 0.02%  Tyrosine: 0.04%  Phenylalanine: 0.03%  Total Lysine: 0.01%  Histidine: 0.01%  Arginine: 0.05%  Proline: 0.05% |
| Cystine & Methionine by Performic Acid Oxidation | MET-LC-005 | % | Cystine: 0.01%  Methionine: 0.01% |
| Tryptophan by Alkaline Hydrolysis | MET-LC-024 | % | 0.01% |
| Vitamin E  (α-tocopherol, β-tocopherol, δ- tocopherol, γ-tocopherol) | MET-VT-009 /MET-VT-030 | mg/100g | 0.1 mg/100g* |
| Vitamin K1 | MET-VT-028 | mg/100g | 0.000625 mg/100g |
| Biotin | MET-VT-003 | mg/100g | 0.0037 mg/100g |
| Folic Acid | MET-VT-018 | mg/100g | 0.0033 mg/100g |
| Vitamin B3 – Niacin | MET-VT-005 | mg/100g | 0.22 mg/100g |
| Vitamin B5 – Panothenic Acid | MET-VT-007 | mg/100g | 0.055 mg/100g |
| Vitamin B6 – Pyridoxine | MET-VT-006 | mg/100g | 0.01 mg/100g |
| Vitamin B2 – Riboflavin | MET-VT-002 | mg/100g | 0.1 mg/100g |
| Vitamin B1 – Thiamin | MET-VT-019 | mg/100g | 0.011 mg/100g |
| Choline | MET-VT-031 | mg/100g | 1 mg/100g |
| Phenolic Acids | MET-LC-004 | Sinapine (%) µg/g (ppm) | Sinapine: 0.05% Ferulic acid: 10 µg/g Coumaric acid: 10 µg/g |
| Glucosinolates | MET-LC-026 | µmol/g | 0.05 µmol/g* |
| Tannins – Soluble Condensed | MET-AN-012 | % | 0.05% |
| Phytic acid | MET-EL-011 | % | 0.14% |
| Calcium | MET-EL-002/MET-EL-003 | % | 0.004% |
| Phosphorus | MET-EL-002/MET-EL-003 | % | 0.004% |
| Magnesium | MET-EL-002/MET-EL-003 | % | 0.001% |
| Potassium | MET-EL-002/MET-EL-003 | % | 0.004% |
| Sodium | MET-EL-002/MET-EL-003 | % | 0.002% |
| Iron | MET-EL-002/MET-EL-003 | % | 0.0002% |
| Zinc | MET-EL-002/MET-EL-003 | % | 0.001% |
| Copper | MET-EL-002/MET-EL-003 | % | 0.0001% |
| Manganese | MET-EL-002/MET-EL-003 | % | 0.00005% |
| Sulfur | MET-EL-009 | % | 0.02% |
| Molybdenum | MET-EL-002/MET-EL-004 | % | 0.00012% |
| Chloride | MET-CM-018 | % | 0.06% |
| Phytosterols | MET-LI-034 | % | 0.002%* |
| Fatty Acid Profile | MET-LI-002/MET-LI-025 | % | C16:0: 0.02%  All others at 0.01% |

**Table B.** Analyte specifics for canola meal compositional analysis

| Parameter | Eurofins Method | Units | LOQ |
| --- | --- | --- | --- |
| Moisture | MET-PR-005 | % | 0.2% |
| Protein, Crude | MET-PR-002 | % | 0.1% |
| Fat, Crude | MET-LI-001 | % | 0.1% |
| Ash | MET-PR-004 | % | 0.4% |
| Carbohydrates, Calculated | OPS-024 | % | Not applicable |
| Crude Fiber | MET-PR-003 | % | 0.2% |
| Acid Detergent Fiber | MET-PR-007 | % | 0.3% |
| Neutral Detergent Fiber | MET-PR-008 | % | 0.3% |
| Amino Acids by Acid Hydrolysis | MET-LC-006 | % | Serine, Glutamic Acid, Glycine, Alanine, Histidine, Total Lysine: 0.01%  Aspartic Acid, Threonine, Valine,  Isoleucine, Leucine: 0.02%  Tyrosine: 0.04%  Phenylalanine: 0.03%  Arginine, Proline: 0.05% |
| Cystine & Methionine by Performic Acid Oxidation | MET-LC-005 | % | Cystine: 0.01%  Methionine: 0.01% |
| Tryptophan by Alkaline Hydrolysis | MET-LC-024 | % | 0.01% |
| Vitamin E  (α-tocopherol, β-tocopherol, δ- tocopherol, γ-tocopherol) | MET-VT-009 /MET-VT-030 | mg/100g | 0.1 mg/100g* |
| Phenolic Acids | MET-LC-004 | Sinapine (%) µg/g (ppm) | Sinapine: 0.05% Ferulic acid: 10 µg/g Coumaric acid: 10 µg/g |
| Glucosinolates | MET-LC-026 | µmol/g | 0.05 µmol/g* |
| Tannins – Soluble Condensed | MET-AN-012 | % | 0.05% |
| Phytic acid | MET-EL-011 | % | 0.14% |
| Calcium | MET-EL-002/MET-EL-003 | % | 0.004% |
| Phosphorus | MET-EL-002/MET-EL-003 | % | 0.004% |
| Phytosterols | MET-LI-034 | % | 0.002%* |
| Fatty Acid Profile | MET-LI-002/MET-LI-025 | % | C16:0: 0.02%  All others at 0.01% |

- Listed LOQ applies to all analyte parameters.

Table C. Glucosinolates of DHA canola in grain (µmol/g DW)

| Analyte | Test  Material | Mean | Std Dev | Min | Max | Reference Range | ILSI db values*  Range |
| --- | --- | --- | --- | --- | --- | --- | --- |
| Epiprogoitrin | Parental control | 0.094 | 0.052 | <LOQ | 0.200 | <LOQ-0.300 | 0.07-0.53 |
|  | DHA canola | 0.096 | 0.053 | <LOQ | 0.200 |  |  |
| Glucoalyssin | Parental control | 0.349 | 0.147 | 0.062 | 0.730 | <LOQ-1.800 | 0.07-0.56 |
|  | DHA canola | 0.363 | 0.150 | 0.130 | 0.670 |  |  |
| Glucobrassicanapin | Parental control | 0.311 | 0.155 | 0.073 | 0.680 | <LOQ-1.300 | 0.39-1.80 |
|  | DHA canola | 0.250 | 0.121 | 0.061 | 0.530 |  |  |
| Glucobrassicin | Parental control | 0.205 | 0.055 | <LOQ | 0.280 | 0.090-0.550 | 0.06-1.84 |
|  | DHA canola | 0.282 | 0.073 | 0.081 | 0.420 |  |  |
| Gluconapin | Parental control | 2.166 | 0.723 | 0.664 | 3.650 | 0.417-6.390 | 0.10-6.84 |
|  | DHA canola | 1.972 | 0.681 | 0.627 | 3.510 |  |  |
| Gluconasturtin | Parental control | 0.094 | 0.052 | <LOQ | 0.180 | <LOQ-0.520 | 0.13-1.65 |
|  | DHA canola | 0.135 | 0.084 | <LOQ | 0.380 |  |  |
| Progoitrin | Parental control | 4.914 | 1.895 | 0.933 | 8.680 | 0.838-17.000 | 0.11-9.73 |
|  | DHA canola | 4.936 | 1.874 | 1.590 | 9.120 |  |  |
| 4-Hydroxyglucobrassicin | Parental control | 3.938 | 0.769 | 1.360 | 5.220 | <LOQ-5.540 | 0.05-10.40 |
|  | DHA canola | 3.849 | 0.964 | 1.120 | 5.730 |  |  |

*ILSI composition database (db), Version 6

Table D. Phytosterols & phenolics of DHA canola and Parental control in grain (%DW), except where noted

| Analyte | Test  Material | Mean | Std Dev | Min | Max | Reference Range | ILSI db values*  Range |
| --- | --- | --- | --- | --- | --- | --- | --- |
| Brassicasterol | Parental control | 0.112 | 0.005 | 0.097 | 0.120 | 0.045-0.170 | 0.006-0.048 |
|  | DHA canola | 0.052 | 0.004 | 0.045 | 0.066 |  |  |
| Campesterol | Parental control | 0.287 | 0.010 | 0.268 | 0.310 | 0.226-0.397 | 0.02-0.13 |
|  | DHA canola | 0.385 | 0.018 | 0.352 | 0.425 |  |  |
| Cholesterol | Parental control | 0.002 | 0.002 | <LOQ | 0.006 | <LOQ-0.050 | 0.0004-0.0028 |
|  | DHA canola | 0.002 | 0.003 | <LOQ | 0.020 |  |  |
| Clerosterol | Parental control | 0.006 | 0.000 | 0.005 | 0.007 | 0.004-0.006 | NR |
|  | DHA canola | 0.006 | 0.000 | 0.006 | 0.007 |  |  |
| Delta-5-avenasterol | Parental control | 0.036 | 0.006 | 0.026 | 0.046 | 0.008-0.037 | 2.5-6.6%  of total sterols** |
|  | DHA canola | 0.044 | 0.008 | 0.030 | 0.064 |  |  |
| Delta-7-avenasterol | Parental control | 0.003 | 0.000 | 0.002 | 0.003 | 0.002-0.004 | ND-0.8%  of total sterols** |
|  | DHA canola | 0.004 | 0.000 | 0.003 | 0.005 |  |  |
| Glucoalyssin | Parental control | 0.349 | 0.147 | 0.062 | 0.730 | <LOQ-1.800 | 0.07-0.56 |
|  | DHA canola | 0.363 | 0.150 | 0.130 | 0.670 |  |  |
| Glucobrassicanapin | Parental control | 0.311 | 0.155 | 0.073 | 0.680 | <LOQ-1.300 | 0.39-1.80 |
|  | DHA canola | 0.250 | 0.121 | 0.061 | 0.530 |  |  |
| Glucobrassicin | Parental control | 0.205 | 0.055 | <LOQ | 0.280 | 0.090-0.550 | 0.06-1.84 |
|  | DHA canola | 0.282 | 0.073 | 0.081 | 0.420 |  |  |
| Gluconapin | Parental control | 2.166 | 0.723 | 0.664 | 3.650 | 0.417-6.390 | 0.10-6.84 |
|  | DHA canola | 1.972 | 0.681 | 0.627 | 3.510 |  |  |
| Gluconasturtin | Parental control | 0.094 | 0.052 | <LOQ | 0.180 | <LOQ-0.520 | 0.13-1.65 |
|  | DHA canola | 0.135 | 0.084 | <LOQ | 0.380 |  |  |
| Sitosterol | Parental control | 0.551 | 0.028 | 0.501 | 0.616 | 0.346-0.580 | 0.03-0.21 |
|  | DHA canola | 0.579 | 0.036 | 0.512 | 0.650 |  |  |
| Stigmasterol | Parental control | 0.003 | <LOQ | 0.002 | 0.004 | <LOQ-0.005 | 0.0010-0.0078 |
|  | DHA canola | <LOQ | 0.001 | <LOQ | 0.006 |  |  |
| 24-Methylene cholesterol | Parental control | 0.013 | 0.005 | 0.008 | 0.020 | 0.003-0.020 | NR |
|  | DHA canola | 0.011 | 0.004 | 0.007 | 0.020 |  |  |
| Delta-5,24-Stigmastadienol | Parental control | 0.007 | 0.001 | 0.006 | 0.008 | 0.003-0.009 | NR |
|  | DHA canola | 0.009 | 0.001 | 0.008 | 0.010 |  |  |
| Delta-7-Stigmastenol | Parental control | 0.002 | 0.000 | 0.002 | 0.002 | 0.002-0.006 | ND-1.3%  of total sterols** |
|  | DHA canola | 0.003 | 0.001 | 0.002 | 0.005 |  |  |
| Total Phytosterols (%DW) | Parental control | 1.025 | 0.040 | 0.966 | 1.118 | 0.702-1.097 | 0.06-0.39 |
|  | DHA canola | 1.106 | 0.061 | 1.013 | 1.249 |  |  |
| Total Phytosterols (%FW) | Parental control | 0.943 | 0.036 | 0.888 | 1.027 | 0.647-1.008 | 0.45-1.13%  of oil** |
|  | DHA canola | 1.014 | 0.054 | 0.932 | 1.142 |  |  |
| *p-*Coumaric Acid (ppm) | Parental control | 19.19 | 4.35 | 12.56 | 30.51 | 10.85-26.65 | NR |
|  | DHA canola | 10.930 | NR | NR | NR |  |  |
| Ferulic Acid (ppm) | Parental control | 137.238 | 23.680 | 101.10 | 184.60 | 88.91-217.50 | NR |
|  | DHA canola | 130.084 | 20.960 | 98.72 | 171.70 |  |  |
| Phytic Acid (ppm) | Parental control | 1.92 | 0.43 | 1.10 | 2.70 | 0.84-2.50 | 0.94-3.88 |
|  | DHA canola | 1.90 | 0.44 | 1.10 | 2.70 |  |  |
| Sinapine  (% DW) | Parental control | 1.264 | 0.078 | 1.089 | 1.415 | 0.876-1.463 | 0.19-1.36 |
|  | DHA canola | 1.191 | 0.070 | 1.031 | 1.330 |  |  |

*ILSI composition database (db), Version 6; **OECD, 2011; NR = Not Reported

Table E. Amino acids of DHA canola in grain (%DW)

| Analyte | Test  Material | Mean | Std Dev | Min | Max | REF Range | ILSI db values*  Range |
| --- | --- | --- | --- | --- | --- | --- | --- |
| Alanine | Parental control | 1.239 | 0.049 | 1.130 | 1.360 | 0.999-1.340 | 0.733-1.430 |
|  | DHA canola | 1.268 | 0.046 | 1.160 | 1.350 |  |  |
| Arginine | Parental control | 1.923 | 0.092 | 1.700 | 2.090 | 1.480-2.090 | 0.969-2.102 |
|  | DHA canola | 1.919 | 0.087 | 1.720 | 2.060 |  |  |
| Aspartic Acid | Parental control | 2.164 | 0.106 | 1.920 | 2.350 | 1.680-2.420 | 1.150-2.623 |
|  | DHA canola | 2.282 | 0.097 | 2.070 | 2.440 |  |  |
| Cystine | Parental control | 0.754 | 0.037 | 0.680 | 0.840 | 0.580-0.820 | 0.189-0.959 |
|  | DHA canola | 0.743 | 0.038 | 0.630 | 0.820 |  |  |
| Glutamic Acid | Parental control | 5.681 | 0.258 | 5.090 | 6.210 | 4.360-6.170 | 3.270-7.310 |
|  | DHA canola | 5.599 | 0.269 | 4.930 | 6.030 |  |  |
| Glycine | Parental control | 1.519 | 0.062 | 1.380 | 1.660 | 1.240-1.660 | 0.856-1.750 |
|  | DHA canola | 1.584 | 0.061 | 1.440 | 1.690 |  |  |
| Histidine | Parental control | 0.843 | 0.032 | 0.774 | 0.910 | 0.677-0.922 | 0.471-1.050 |
|  | DHA canola | 0.845 | 0.036 | 0.755 | 0.900 |  |  |
| Isoleucine | Parental control | 1.218 | 0.052 | 1.080 | 1.320 | 0.931-1.310 | 0.649-1.350 |
|  | DHA canola | 1.218 | 0.048 | 1.100 | 1.290 |  |  |
| Leucine | Parental control | 2.129 | 0.092 | 1.890 | 2.300 | 1.660-2.300 | 1.140-2.350 |
|  | DHA canola | 2.120 | 0.086 | 1.920 | 2.280 |  |  |
| Lysine | Parental control | 1.890 | 0.107 | 1.670 | 2.130 | 1.490-2.140 | 1.070-2.090 |
|  | DHA canola | 1.948 | 0.129 | 1.730 | 2.240 |  |  |
| Methionine | Parental control | 0.611 | 0.023 | 0.570 | 0.660 | 0.490-0.670 | 0.191-0.705 |
|  | DHA canola | 0.623 | 0.027 | 0.560 | 0.660 |  |  |
| Phenyl-  alanine | Parental control | 1.217 | 0.054 | 1.080 | 1.320 | 0.949-1.310 | 0.694-1.520 |
|  | DHA canola | 1.202 | 0.046 | 1.100 | 1.290 |  |  |
| Proline | Parental control | 1.925 | 0.086 | 1.700 | 2.110 | 1.460-2.050 | 1.010-2.130 |
|  | DHA canola | 1.865 | 0.091 | 1.670 | 2.090 |  |  |
| Serine | Parental control | 1.279 | 0.050 | 1.150 | 1.370 | 1.020-1.380 | 0.662-1.530 |
|  | DHA canola | 1.292 | 0.051 | 1.180 | 1.390 |  |  |
| Threonine | Parental control | 1.280 | 0.044 | 1.170 | 1.380 | 1.040-1.360 | 0.717-1.380 |
|  | DHA canola | 1.318 | 0.045 | 1.220 | 1.400 |  |  |
| Tyrosine | Parental control | 0.789 | 0.035 | 0.702 | 0.854 | 0.644-0.839 | 0.414-0.926 |
|  | DHA canola | 0.817 | 0.029 | 0.756 | 0.878 |  |  |
| Tryptophan | Parental control | 0.456 | 0.020 | 0.410 | 0.500 | 0.340-0.490 | 0.165-0.442 |
|  | DHA canola | 0.453 | 0.021 | 0.400 | 0.500 |  |  |
| Valine | Parental control | 1.562 | 0.063 | 1.400 | 1.690 | 1.160-1.650 | 0.817-1.700 |
|  | DHA canola | 1.566 | 0.068 | 1.420 | 1.680 |  |  |

* ILSI composition database (db), Version 6

Table F. Minerals of DHA canola in grain (ppm DW)

| Analyte | Test  Material | Nº | Mean  (ppm DW) | Std Dev | Min | Max | Reference Range | ILSI db values*  Range |
| --- | --- | --- | --- | --- | --- | --- | --- | --- |
| Calcium | Parental control | 39 | 3,563 | 613 | 2,230 | 4,640 | 2,040-  4,880 | 2,480-14,100 |
|  | DHA canola | 40 | 3,116 | 484 | 2,060 | 3,950 |  |  |
| Copper | Parental control | 39 | 2 | 1 | 1 | 4 | 2-20 | 1.13-9.84 |
|  | DHA canola | 40 | 3 | 3 | 1 | 20 |  |  |
| Iron | Parental control | 39 | 55 | 8 | 40 | 70 | 40-80 | 34.2-843.9 |
|  | DHA canola | 40 | 68 | 10 | 50 | 90 |  |  |
| Magnesium | Parental control | 39 | 3,081 | 211 | 2,610 | 3,510 | 2,460-3,700 | 2,210.3-5,310.1 |
|  | DHA canola | 40 | 3,077 | 210 | 2,620 | 3,510 |  |  |
| Manganese | Parental control | 39 | 32 | 6 | 20 | 40 | 20-40 | 15.45-108.1 |
|  | DHA canola | 40 | 32 | 7 | 20 | 50 |  |  |
| Phosphorus | Parental control | 39 | 6,549 | 1224 | 4,180 | 8,820 | 3,650-8,690 | 4,080.0-18,500.0 |
|  | DHA canola | 40 | 6,686 | 1234 | 4,370 | 8,860 |  |  |
| Potassium | Parental control | 39 | 6655 | 0929 | 4,850 | 8,660 | 5,320-9,150 | 4,610.0-14,000.0 |
|  | DHA canola | 40 | 7816 | 0816 | 6,210 | 9,680 |  |  |
| Sodium | Parental control | 39 | 25 | 14 | 20 | 50 | 20-100 | 1.419-1,360.000 |
|  | DHA canola | 40 | 25 | 16 | 20 | 70 |  |  |
| Sulfur | Parental control | 39 | 5121 | 309 | 4,400 | 5,700 | 3,800-6,500 | 3,698.9-8,896.4 |
|  | DHA canola | 40 | 5100 | 332 | 4,300 | 5,800 |  |  |
| Zinc | Parental control | 39 | 43 | 7 | 30 | 60 | 30-60 | 22.2-154.6 |
|  | DHA canola | 40 | 46 | 8 | 30 | 60 |  |  |

*ILSI composition database (db), Version 7

Table G. Vitamins of DHA canola in grain (mg/100g DW, except where noted)

| Analyte | Test  Material | Mean | Std Dev | Min | Max | Reference Range | ILSI db*  Mean  (Range) |
| --- | --- | --- | --- | --- | --- | --- | --- |
| Alpha Tocopherol  Vitamin E | Parental control | 11.94 | 6.61 | 9.17 | 51.70 | 10.90-31.30 | 0.957-17.962 |
|  | DHA canola | 15.69 | 5.78 | 12.40 | 49.70 |  |  |
| Beta Tocopherol  Vitamin E | Parental control | 0.163 | 0.087 | 0.182 | 3.350 | 0.108-0.649 | 0.133-0.288 |
|  | DHA canola | 0.142 | 0.027 | 0.111 | 0.236 |  |  |
| Biotin | Parental control | 0.055 | 0.004 | 0.047 | 0.066 | 0.047-0.088 | NR |
|  | DHA canola | 0.069 | 0.049 | 0.059 | 0.083 |  |  |
| Choline | Parental control | 262.73 | 21.59 | 220.51 | 312.25 | 195.37-381.31 | NR |
|  | DHA canola | 276.05 | 23.33 | 229.14 | 328.40 |  |  |
| Delta Tocopherol  Vitamin E | Parental control | 0.456 | 0.526 | 0.182 | 3.350 | 0.110-13.500 | 0.143-1.510 |
|  | DHA canola | 0.283 | 0.088 | 0.112 | 0.567 |  |  |
| Vitamin B9  (Folic Acid) | Parental control | 0.123 | 0.036 | 0.09 | 0.23 | 0.035-0.634 | 0.0752-0.8750 |
|  | DHA canola | 0.123 | 0.032 | 0.041 | 0.212 |  |  |
| Gamma Tocopherol  Vitamin E | Parental control | 21.21 | 1.83 | 17.70 | 25.00 | 10.20-72.20 | 2.50-27.40 |
|  | DHA canola | 22.78 | 1.92 | 17.80 | 26.20 |  |  |
| Niacin  Vitamin B3 | Parental control | 9.66 | 0.96 | 7.89 | 11.50 | 8.41-16.80 | 2.92-26.10 |
|  | DHA canola | 15.14 | 1.91 | 10.60 | 18.90 |  |  |
| Pantothenic Acid  Vitamin B5 | Parental control | 0.46 | 0.10 | 0.22 | 0.81 | 0.20-0.82 | 0.37-2.57 |
|  | DHA canola | 0.56 | 0.11 | 0.34 | 0.75 |  |  |
| Pyridoxine  Vitamin B6 | Parental control | 0.54 | 0.06 | 0.45 | 0.68 | 0.44-0.98 | 0.175-1.330 |
|  | DHA canola | 0.85 | 0.10 | 0.63 | 1.10 |  |  |
| Riboflavin  Vitamin B2 | Parental control | 0.32 | 0.06 | 0.26 | 0.58 | 0.20-0.58 | 0.193-1.040 |
|  | DHA canola | 0.35 | 0.03 | 0.29 | 0.43 |  |  |
| Thiamin  Vitamin B1 | Parental control | 1.29 | 0.20 | 0.79 | 1.71 | 0.19-2.27 | 0.334-2.040 |
|  | DHA canola | 1.48 | 0.23 | 1.05 | 1.95 |  |  |
| Total Tocopherols  Vitamin E | Parental control | 33.68 | 7.20 | 28.60 | 75.10 | 24.50-96.90 | 3.582-38.939 |
|  | DHA canola | 38.88 | 5.90 | 31.20 | 71.00 |  |  |
| Vitamin K1  (mg/kg) | Parental control | 0.489 | 0.065 | 0.372 | 0.626 | 0.252-0.669 | 0.40-5.63 |
|  | DHA canola | 0.533 | 0.055 | 0.412 | 0.651 |  |  |

NR = Not Reported; *ILSI composition database (db), Version 6

**Table H.**  Putative ORF analysis of 30 or more amino acids across 62,000 bp of inserted DNA (start-to-stop approach)

| ORF  Search^1^ | Length ORF / Aligned AA | Allergen *E* score | Percent identity | Accession / Gene Source |  |
| --- | --- | --- | --- | --- | --- |
| AO2 Chromosome  ORF34  AOL | 114/96 | 7.8e-5 | 38.5% | 31321942  2S albumin *Juglans nigra* |  |
| AO2 Chromosome ACR53360.1  BLASTP Protein  (Pyrco-d5E) | Allergen  239 AA | 9.4e-25 | 33% | XP_005650877  Genome *algae* |  |
|  | No keyword  267 AA | 0.0  (<1e-100) | 100% | ACR53360  *Coccomyxa subellipsoidea* C-169 |  |
| AO2 Chromosome A4KDP0.1  BLASTP Protein  (Pavsa-d5D) | Toxin  41 AA | 0.002 | 41% | KKO86649  C*orynebacterium* |  |
|  | Toxic  26 AA | 9.8 | 26% | KNG44469  *Stemphylium* |  |
|  | No keyword  425 AA | 0 | 100% | A4KDP0  *Rebecca salina* |  |
| AO2 Chromosome XP_002494184  BLASTP Protein  (Picpa-w3D) | Allergen  93 AA | 2.3 | 23% | ACU89247  *Desulfomicrobium* |  |
|  | Toxin  68 AA | 0.071 | 32% | EDX6501*0*  *Bacillus cereus* |  |
|  | Toxic  50 AA | 0.028 | 40% | KHQ50621  M*ameliella* |  |
|  | No keyword  415 AA | 0 | 100% | XP_002494184  *Komagataella phaffii* |  |
| AO2 Chromosome XP_003056992  BLASTP Protein  (Micpu-d6D) | Allergen  38 AA | 4.2 | 47.4% | CCX31489  *Pyronema omphalodes* |  |
|  | Toxin  45 AA | 0.01 | 40% | KKO86649  *Corynebacterium ulcerans* |  |
|  | Toxic  41 AA | 7e-4 | 41% | ODM29929  *Marinobacter adhaerens* |  |
|  | No keyword  463 AA | 0 | 100% | XP_003056992  *Micromonas pusilla* |  |
| AO5 Chromosome  AOL^3^ | 114/96 | 7.8e-5 | 38.5% | AAM54365  2S albumin *Juglans nigra* |  |
| AO5 Chromosome  ACR53360  BLASTP Protein  (Pyrco-d5E) | Allergen  239 AA | 9.4e-25 | 33% | XP_005650877  *Coccomyxa subellipsoidea* |  |
|  | No keyword  267 AA | 0 | 100% | ACR53360  *Pyramimonas cordata* |  |
| AO5 Chromosome  ACR53359  BLASTP Protein  (Pyrco-d6E) | Allergen  225 AA | 1.6e-37 | 3.9% | XP_005650877  *Coccomyxa subellipsoidea* |  |
|  | Toxin  69 AA | 3.7 | 31.9% | 4RGN_B  Mab connected to Staph Enterotoxin B |  |
|  | No keyword  288 AA | 0 | 100% | ACR53359  *Pyramimonas cordata* |  |
| AO5 Chromosome  WP_003988626  BLASTP Protein  (PAT) | Allergen  25 AA | 9.3 | 64% | AEV97129  *Niastella koreensis* |  |
|  | Toxin  161 AA | 2.4e-29 | 42.8% | CCK25597  *Streptomyces davaonensis* |  |
|  | Toxic  168 AA | 8.4e-105 | 85.7% | AXS75741  p390-blpR-cmcas9-gfp^2^ |  |
|  | No keyword | 0 | 100% | WP_003988626  *Streptomyces viridochromogenes* |  |
| AO5 Chromosome  XP_002494184  BLASTP Protein  (Picpa-w3D) | Allergen  93 AA | 2.3 | 22.6% | ACU89247  *Desulfomicrobium baculatum* |  |
|  | Toxin  68 AA | 0.071 | 32.3% | EDX65010  *Bacillus cereus* |  |
|  | Toxic  50 AA | 0.028 | 40% | KHQ50621  *Mameliella alba* |  |
|  | No keyword  415 AA | 0 | 100% | XP_002494184  *Komagataella phaffii* |  |
| AO5 chromosome  A4KDP0  BLASTP Protein  (Pavsa-d5D) | Toxin  41 AA | 0.002 | 41.5% | KKO86649  *Corynebacterium ulcerans* |  |
|  | Toxic  26 AA100% | 9.8 | 50% | KNG44469  *Stemphylium lycopersici* |  |
|  | No Keyword  425 AA | 0 | 100% | A4KDP0  *Pavlova salina* |  |
| AO5 chromosome  A0PJ29  BLASTP Protein  (Pavsa-d4D) | Toxin  290 AA | 2.5e-12 | 28.2% | ANA38154  *Acinetobacter baumannii* |  |
|  | Toxic  56 AA | 1.3 | 32.1% | KEI69057  *Planktothrix agardhii* |  |
|  | No keyword  447 AA | 0 | 100% | A0PJ29  *Rebecca salina* |  |
| AO5 chromosome  BAD08375  BLASTP Protein  (Lackl-d12D) | Toxin  58 AA | 0.1 | 27.6% | KKC53285  *Bacillus sp.* |  |
|  | Toxic  54 AA | 4.8e-4 | 33.3% | KEJ96575  *Sulfitobacter pseudonitzschiae* |  |
|  | No Keyword  416 AA | 0 | 100% | BAD08375  *Lachancea kluyveri* |  |
| AO5 Chromosome  XP_003056992  BLASTP Protein  (Micpu-d6D) | Allergen  38 AA | 4.2 | 47.4% | CCX31489  *Pyronema omphalodes* |  |
|  | Toxin  45 AA | 0.01 | 40% | KKO86649  *Corynebacterium ulcerans* |  |
|  | Toxic  41 AA | 7e-4 | 43.9% | ODM29929  *Marinobacter adhaerens* |  |
|  | No keyword  463 AA | 0 | 100% | XP_003056992  *Micromonas pusilla* | |

^1^AOL FASTA or BLASTP Protein

^2^Expression vector

^3^ Identical segments
